# Predicting acute pancreatitis severity with enhanced computed tomography scans using convolutional neural networks

**DOI:** 10.1038/s41598-023-44828-7

**Published:** 2023-10-16

**Authors:** Hongyin Liang, Meng Wang, Yi Wen, Feizhou Du, Li Jiang, Xuelong Geng, Lijun Tang, Hongtao Yan

**Affiliations:** 1 Department of General Surgery, The General Hospital of Western Theater Command (Chengdu Military General Hospital), Chengdu, 610083 China; 2 Sichuan Provincial Key Laboratory of Pancreatic Injury and Repair, Chengdu, 610083 China; 3 Department of Traditional Chinese Medicine, The General Hospital of Western Theater Command (Chengdu Military General Hospital), Chengdu, 610083 China; 4 Department of Radiology, The General Hospital of Western Theater Command (Chengdu Military General Hospital), Chengdu, 610083 China; 5 Department of Cardiac Surgery, The General Hospital of Western Theater Command (Chengdu Military General Hospital), Chengdu, 610083 China; 6 Department of Liver Transplantation and Hepato-biliary-pancreatic Surgery, Sichuan Cancer Hospital & Institute, Sichuan Cancer Center, School of Medicine, University of Electronic Science and Technology of China, Chengdu, 610016 China

**Keywords:** Computational models, Machine learning, Predictive medicine, Gastrointestinal diseases, Pancreatitis, Image processing

## Abstract

This study aimed to evaluate acute pancreatitis (AP) severity using convolutional neural network (CNN) models with enhanced computed tomography (CT) scans. Three-dimensional DenseNet CNN models were developed and trained using the enhanced CT scans labeled with two severity assessment methods: the computed tomography severity index (CTSI) and Atlanta classification. Each labeling method was used independently for model training and validation. Model performance was evaluated using confusion matrices, areas under the receiver operating characteristic curve (AUC-ROC), accuracy, precision, recall, F1 score, and respective macro-average metrics. A total of 1,798 enhanced CT scans met the inclusion criteria were included in this study. The dataset was randomly divided into a training dataset (n = 1618) and a test dataset (n = 180) with a ratio of 9:1. The DenseNet model demonstrated promising predictions for both CTSI and Atlanta classification-labeled CT scans, with accuracy greater than 0.7 and AUC-ROC greater than 0.8. Specifically, when trained with CT scans labeled using CTSI, the DenseNet model achieved good performance, with a macro-average F1 score of 0.835 and a macro-average AUC-ROC of 0.980. The findings of this study affirm the feasibility of employing CNN models to predict the severity of AP using enhanced CT scans.

## Introduction

Acute pancreatitis (AP) is a common acute abdominal disease in clinical practice^1^. Mild acute pancreatitis (MAP) has a good prognosis, while severe acute pancreatitis (SAP) is often associated with complications such as pancreatic necrosis and organ failure, resulting in a high mortality rate^2^. As the clinical course of AP strongly depends on the early management of the disease, accurate assessment of the severity of AP can facilitate early intervention and contribute to improved clinical outcomes^3, 4^.

Several assessment systems that utilize clinical manifestations, laboratory tests have been developed and evaluated in predicting the severity and prognosis of AP, including the Acute Physiology and Chronic Health Evaluation (APACHE II)^5^, the Ranson system^6^, the Bedside Index for Severity in Acute Pancreatitis (BISAP)^7^, the Marshall score^8^, the Sepsis-related Organ Failure Assessment (SOFA)^9^. However, these systems, either individually or in combination, have not provided satisfactory predictions for SAP^10^. Machine learning and other artificial intelligence methods have shown promise in forecasting the severity of AP^11^. Studies have shown that machine learning models based on patient demographics and biochemical markers can enhance prediction accuracy^12–14^.

In clinical practice, CT scans play a vital role in assessing the severity of pancreatitis. Several CT scan-related assessment systems, such as the Computed Tomography Severity Index (CTSI)^15^, the Modified Computed Tomography Severity Index (MCTSI)^16^, and the extrapancreatic inflammation on computed tomography (EPIC) score^17^, have shown associations with the severity and prognosis of AP. Several radiomics studies have also been applied in the prediction of AP^18, 19^. However, the use of deep learning models based on CT images for evaluating AP severity is still in its early stages. A recent study by Chen et al. constructed a deep learning model based on MobileNetV2 using non-enhanced CT images obtained from AP patients within 72 h after onset^20^. The results demonstrated that the CT image-based deep learning model achieved a prediction accuracy of 72.3% with an AUC-ROC of 0.741 for MAP, and an accuracy of 79.5% with an AUC-ROC of 0.896 for SAP. These findings suggest that using deep learning models with CT scans to predict the severity of AP is feasible and holds great promise for future applications.

Notably, the use of non-enhanced CT images obtained upon admission may be insufficient for a comprehensive evaluation of AP severity. In the initial stages of the disease, the pancreas undergoes rapid morphological changes and necrosis, which may remain undetectable or underestimated in non-enhanced CT scans^21^. Relevant guidelines^22, 23^ recommend that the optimal timing for CT scans used in assessing the severity of AP is at least 72–96 h after the onset of symptoms, and enhanced CT scans should be utilized.

In this study, we developed a convolutional neural network (CNN) model using 3D DenseNet, to predict the severity of AP using enhanced CT scans. Additionally, we investigated two distinct approaches for severity grading of AP, CTSI and the Atlanta classification, to label the enhanced CT scans. The CTSI can be derived solely from CT images, whereas the Atlanta classification, being more commonly used, incorporates factors beyond CT images. Each labeling method was used independently for model training and validation, facilitating a comprehensive comparison of the predictive performance of the models.

## Methods

### Study design

This was a single-center retrospective study conducted in a tertiary care hospital in western China. The study was approved by the Institutional Ethics Committee (No. A20200212008), and a waiver of informed consent was obtained.

CT scan data from an AP database established in 2009 were utilized^24^, comprising enhanced CT scans of patients diagnosed with AP from 2009 to 2022. The database includes 2,571 abdomen-enhanced CT scans from 1,945 patients diagnosed with AP. Exclusions included patients under 18 years old, patients who have undergone retroperitoneal puncture and catheterization, those with chronic pancreatitis, a history of upper abdominal surgery (except cholecystectomy and bile duct exploration), or tumors. Ultimately, 1,798 enhanced CT scans were included in this study.

## Definition

### Diagnosis of AP

A diagnosis of AP was made according to the 2012 revised Atlanta classification and definitions of AP^2^; patients had to meet any two of the following conditions: (1) abdominal pain consistent with the characteristics of AP; (2) serum amylase (or lipase) greater than three times the upper limit of normal; and (3) characteristic findings of AP on imaging.

### Definition of CTSI

Balthazar proposed the CTSI score based on enhanced CT images, considering pancreatic inflammation and the area proportion of pancreatic necrosis^15^. The detailed scoring criteria are provided in Table 1.

**Table 1:** Computed tomography severity index.

Category	Score
Pancreatic inflammatory	
Normal pancreas	0
Focal or diffuse enlargement of the pancreas	1
Intrinsic pancreatic abnormalities and inflammatory changes in the peripancreatic fat	2
Single, ill-defined area of fluid collection	3
Two or multiple, poorly defined area of fluid collections	4
Pancreatic necrosis	
Normal	0
Mildly necrotic (≤ 30%)	2
Moderately necrotic (> 30% and ≤ 50%)	4
Extensively necrotic (> 50%)	6

### Classification of AP severity

In this study, we used two approaches for assessing AP severity. Firstly, classification based on CTSI scores: a total CTSI of 0–3 indicated MAP, 4–6 indicated moderately severe AP (MSAP), and 7–10 indicated SAP. Secondly, the classification based on the 2012 revised Atlanta classification, which also defined three degrees of AP severity, as outlined in Table 2.

**Table 2:** Grades of acute pancreatitis severity based on the 2012 revised Atlanta classification.

Severity grade	
Mild acute pancreatitis	No organ failure
	No local or systemic complications
Moderately severe acute pancreatitis	Organ failure that resolves within 48 h (transient organ failure)
	Local or systemic complications without persistent organ failure
Severe acute pancreatitis	Persistent (single or multiple) organ failure (> 48 h)

## CT scans acquisition and labeling

All patients underwent standard contrast-enhanced abdominal CT examinations using a single-source, 64-multidetector CT scanner. Specific parameters were as follows: slice thickness of 1.0 mm and a matrix size of 512 × 512. CT scans usually consisted of 300–350 slices. Following non-enhanced CT acquisition, enhanced CT images were obtained after intravenous administration of nonionic iodinated contrast material (300 mg/mL of iodine) at a dose of 1.2 mL/kg and an injection rate of 2.5 mL/s using an automatic power injector. CT scans were saved in digital imaging and communications in medicine (DICOM) format to the picture archiving and communication system (PACS).

Portal venous phase CT images (50–70 s after contrast injection) were extracted from the PACS and used in this study. The CT image window width was adjusted to 200, and the window position was set at 45. Raw Hounsfield unit (HU) values were rescaled to a range of 0 to 1. Each CT scan included 256 manually selected slices encompassing the pancreas, and the CT scan image size was reshaped to 64 × 128 × 128.

CT scans were labeled for AP severity based on CTSI or the 2012 revised Atlanta classification. CTSI scores were determined by radiologists (Du and Geng, each with more than 10 years of experience) using the CTSI criteria according to the CTSI criteria. The radiologists were blinded to patient clinical symptoms and treatment. AP severity based on CTSI was determined by the calculated CTSI score, while severity based on the Atlanta classification was extracted from the database, recorded during patient hospitalization. These data were classified into MAP, MSAP, and SAP groups accordingly. Notably, the AP severity classification based on CTSI did not exactly match that based on the Atlanta classification. Each labeling method was used independently for model training and validation.

## Model development and evaluation

### Training and test datasets

The 1,798 CT scans were randomized into the training dataset (n = 1,618) and the test dataset (n = 180) at a ratio of 9:1. To enhance the training process, data augmentation techniques such as random rotation and translation were applied to the CT scans. As the CT scans were represented as rank-3 shape tensors (samples, depth, height, width), an additional dimension of size 1 at axis 4 was added to enable 3D convolutions (samples, depth, height, width, 1). The training dataset included both the raw CT scans and augmented CT scans, while only the raw CT scans were used for model evaluation in the test dataset.

### DenseNet model

**Figure 1:**
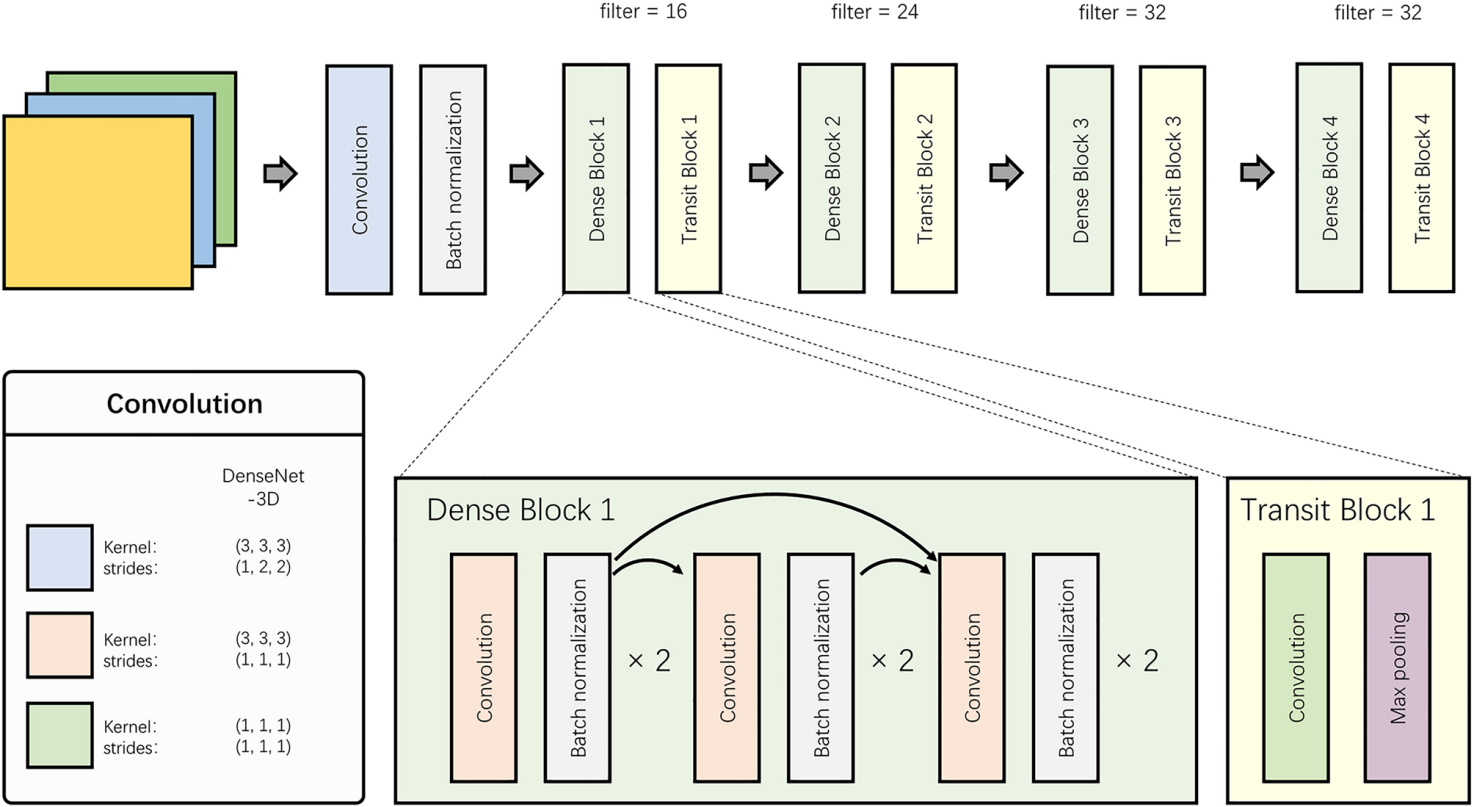
Network architecture of DenseNet.

A three-dimensional DenseNet CNN model was developed for this study, utilizing the network architecture presented in Fig. 1. The model consisted of four modules, each comprising a dense block and a transition block. Within the dense block, the output Xi of layer i satisfied expression (1), where the nonlinear transformation function Hi(·) incorporated batch normalization and convolution. The last module connected the fully connected layers and applied the Softmax function to produce the final predictions.

1 $${\text{X}}_{{\text{i}}} = {\text{H}}_{{\text{i}}} \left( {\left[ {{\text{x}}_{0} ,{\text{x}}_{{1}} , \ldots ,{\text{x}}_{{{\text{i}} - {1}}} } \right]} \right).$$

### Model evaluation

Confusion matrices were used to assess the accuracy of pairwise classification between different categories of patients. Since the task involved multiclassification prediction, the metrics such as AUC-ROC, precision, recall, and F1 score were calculated. The macro-average values of these metrics, computed as the arithmetic mean across individual classes, were used to evaluate the model's performance. In this study, macro-average metrics were employed instead of micro-average metrics to evaluate model performance in the triple classification task^25^. Macro-average metrics, which assign equal importance to each class, are considered more suitable for imbalanced datasets compared to micro-average metrics^26^. By giving equal weight to each class, macro-average metrics provide objective results for imbalanced datasets, allowing for reliable evaluation.

### Visual interpretation of the models

The interpretation of model predictions was achieved by employing Gradient-weighted Class Activation Mappings (Grad-CAMs) extended to the 3D setting^27^. These visual explanations represent heat maps superimposed on each slice, providing insights into the model's decision-making process. To visualize the Grad-CAMs, we overlay the Grad-CAMs on each input slice, offering a comprehensive view of the prediction rationale.

## Ethics statement and informed consent statement

The study was approved by the Institutional Ethics Committee of the General Hospital of Western Theater Command (No. A20200212008). The requirement for obtaining written informed consent from patients was waived by the Institutional Ethics Committee of the General Hospital of Western Theater Command due to the retrospective nature of this study. Our study was conducted according to the ethical standards of the 1964 Declaration of Helsinki and its later amendments.

## Methods statement

All methods were carried out in accordance with relevant guidelines and regulations.

## Experimental environment and statistical analysis

This study was conducted on a computer with an NVIDIA(R) RTX(R) 3090 TI GPU and Intel(R) Core(R) CPU i9-12900 K processor. Python 3.9.0 (Python Software Foundation, Wilmington, DE, USA) was used for data extraction and preprocessing, model development and validation, and visualization and statistical analysis. To calculate the Ninety-five percent confidence intervals (CIs) for performance evaluation metrics such as accuracy and F1 score, we implemented bootstrapping with 1,000 iterations^28^. This allowed us to derive values from these iterations, upon which the CIs were computed. Statistical significance was computed with the same bootstrapping method^29^. *P* < 0.05 was considered statistically significant.

## Results

**Figure 2:**
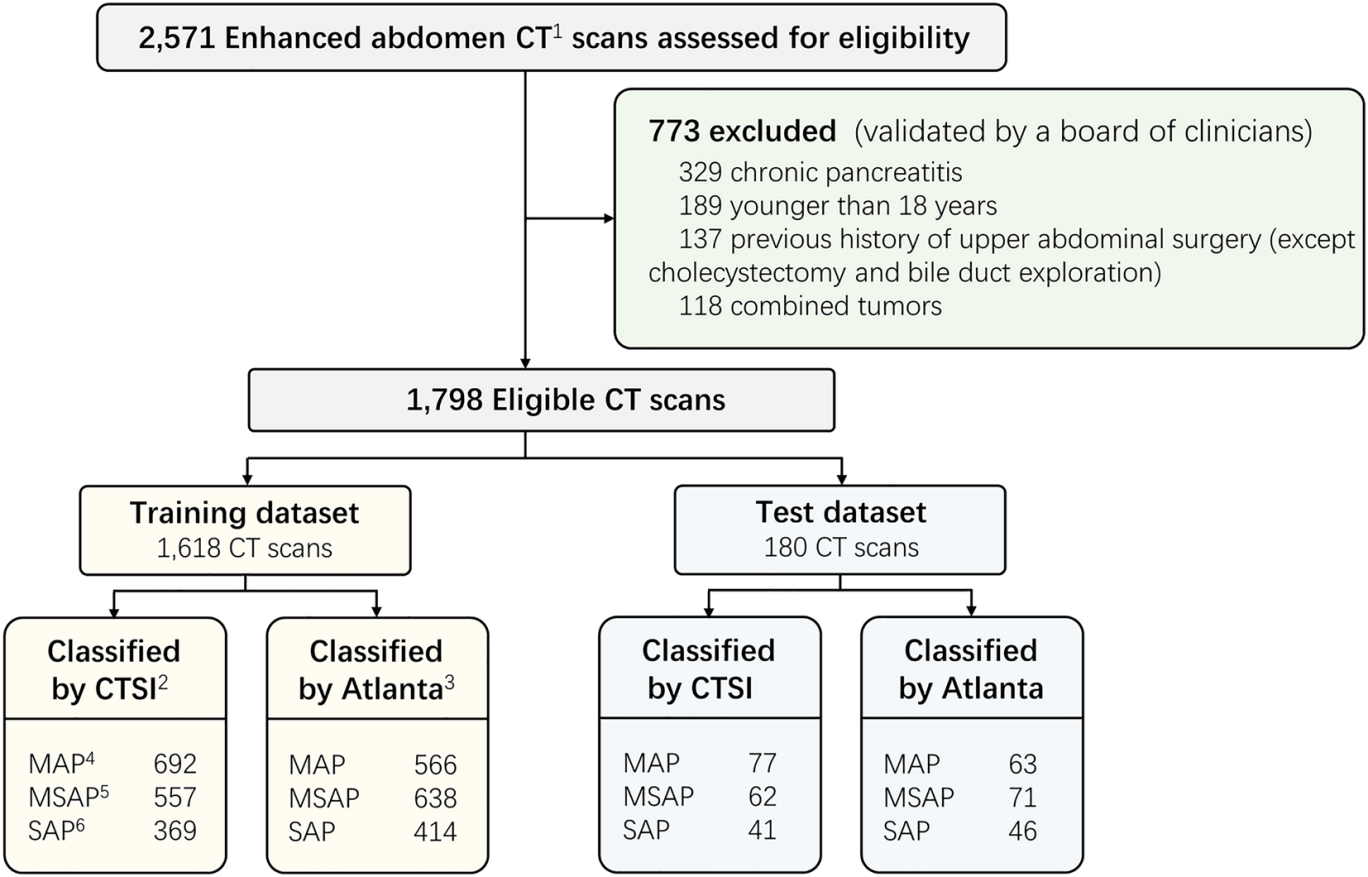
Dataset Selection Flow Diagram. Note: ^1^CT, computed tomography; ^2^CTSI, computed tomography severity index; ^3^Atlanta, 2012 revised Atlanta classification of acute pancreatitis; ^4^MAP, mild acute pancreatitis; ^5^MSAP, moderate severe acute pancreatitis; ^6^SAP, severe acute pancreatitis.

In this study, a total of 1,798 enhanced CT scans from 1,561 patients were included (Fig. 2). These CT scans were labeled according to both the CTSI (MAP: 769, 42.8%; MSAP: 619, 34.4%; SAP: 410, 22.8%) and the 2012 revised Atlanta classification (MAP: 629, 35.0%; MSAP: 709, 39.4%; SAP: 460, 25.6%) to determine the severity of AP. Notably, there were 173 instances (9.6%) where the severity determination based on the CTSI did not correspond with the Atlanta classification. Of these, 154 instances were allocated to the training dataset, and 19 to the test dataset. Specifically, 123 instances categorized as MAP based on the CTSI were classified as MSAP according to the Atlanta classification. 17 instances categorized as MAP by CTSI were later classified as SAP under the Atlanta criteria. Furthermore, 33 instances categorized as MSAP by CTSI were classified as SAP according to the Atlanta classification. The dataset was randomly divided into a training dataset (n = 1,618) and a test dataset (n = 180) with a ratio of 9:1.

The demographic characteristics and clinical outcomes of the patients in both the training and test datasets are summarized in Table 3. There were no significant differences between the two groups in terms of age, gender, etiology, length of hospital stay, and mortality rate (*P* > 0.05). Furthermore, the time interval from the onset of symptoms to the CT examination for both groups was 5.4 ± 0.9 days and 5.4 ± 0.8 days, respectively, demonstrating no significant variance (*P* > 0.05).

**Table 3:** Demographic characteristics and clinical outcomes of the training and validation cohort.

	Training dataset (n = 1,618)	Test dataset (n = 180)	*P*
Age, mean ± SD	49.7 ± 9.4	50.6 ± 9.9	0.201
Sex, male, n(%)	938 (58.0%)	102 (56.7%)	0.797
Etiology			0.976
Hypertriglyceridemia	726 (44.9%)	82 (45.6%)	
Biliary	534 (33.0%)	58 (32.2%)	
Other	358 (22.1%)	40 (22.2%)	
Time interval between CT scan and the onset, days, mean ± SD	5.4 ± 0.9	5.4 ± 0.8	0.488
LOS, days, mean ± SD	10.9 ± 3.1	11.0 ± 2.9	0.554
Mortality, n (%)	76 (4.7%)	7 (3.9%)	0.762

*LOS* length of stay.

*P < 0·05.

**Figure 3:**
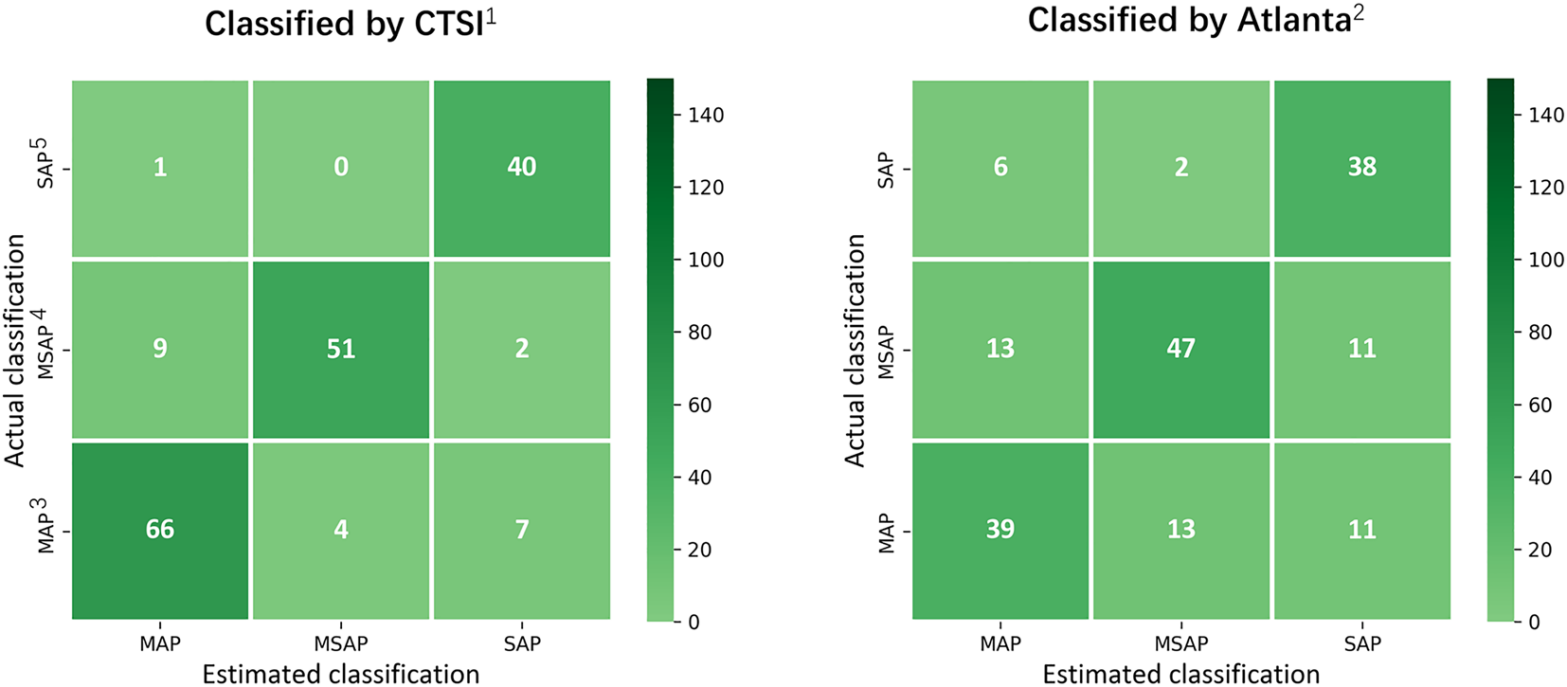
Confusion matrices of the model prediction. The DenseNet model was used to estimate the severity of acute pancreatitis based on (**A**) CTSI-labelled CT scans and (**B**) Atlanta classification-labelled CT scans. Note: ^1^CTSI, computed tomography severity index; ^2^Atlanta, 2012 revised Atlanta classification of acute pancreatitis; ^3^MAP, severe acute pancreatitis; ^4^MSAP, moderately severe acute pancreatitis; and ^5^SAP, severe acute pancreatitis.

The performance of the trained models are summarized in Table 4, and the confusion matrices depicting the prediction results are presented in Fig. 3. The results revealed that the DenseNet model achieved favorable predictions for both the CTSI- and Atlanta classification-labeled CT scans, with accuracy exceeding 0.7 and AUC-ROC exceeding 0.7. Notably, the model trained with CTSI-labeled CT scans demonstrated particularly favorable performance, with a macro-average accuracy of 0.899, macro-average F1 score of 0.835, and macro-average AUC-ROC of 0.980.

**Table 4:** Predictive performance of the models trained using CT scans labeled with CTSI and Atlanta classification.

		Accuracy	Recall	Percision	F1 score	AUC-ROC
Model trained using CT scans labeled with CTSI	MAP, [95% CI]	0.871 [0.813–0.919]	0.773 [0.619–0.881]	0.927 [0.829–1.000]	0.834 [0.750–0.909]	0.967 [0.935–0.990]
	MSAP, [95% CI]	0.883 [0.828–0.935]	0.689 [0.559–0.868]	0.951 [0.846–1.000]	0.781 [0.654–0.878]	0.978 [0.946–0.995]
	SAP, [95% CI]	0.942 [0.899–0.980]	0.948 [0.841–1.000]	0.837 [0.726–0.956]	0.890 [0.824–0.957]	0.993 [0.981–1.000]
	Macro-average, [95% CI]	0.899 [0.847–0.945]	0.803 [0.673–0.916]	0.905 [0.799–0.985]	0.835 [0.742–0.915]	0.980 [0.954–0.995]
Model trained using CT scans labeled with Altanta	MAP, [95% CI]	0.782 [0.711–0.850]	0.523 [0.356–0.686]	0.787 [0.667–0.909]	0.622 [0.453–0.756]	0.843 [0.763–0.904]
	MSAP, [95% CI]	0.779 [0.697–0.838]	0.559 [0.422–0.692]	0.855 [0.722–0.961]	0.669 [0.542–0.777]	0.859 [0.767–0.919]
	SAP, [95% CI]	0.864 [0.793–0.924]	0.714 [0.539–0.881]	0.736 [0.598–0.887]	0.717 [0.563–0.863]	0.890 [0.791–0.962]
	Macro-average, [95% CI]	0.809 [0.733–0.871]	0.599 [0.439–0.753]	0.793 [0.662–0.919]	0.670 [0.519–0.799]	0.864 [0.774–0.928]

*CTSI* computed tomography severity index, *MAP* severe acute pancreatitis, *CI* confidence interval, *MSAP* moderately severe acute pancreatitis, *SAP* severe acute pancreatitis, *Atlanta* 2012 revised Atlanta classification of acute pancreatitis.

**Figure 4:**
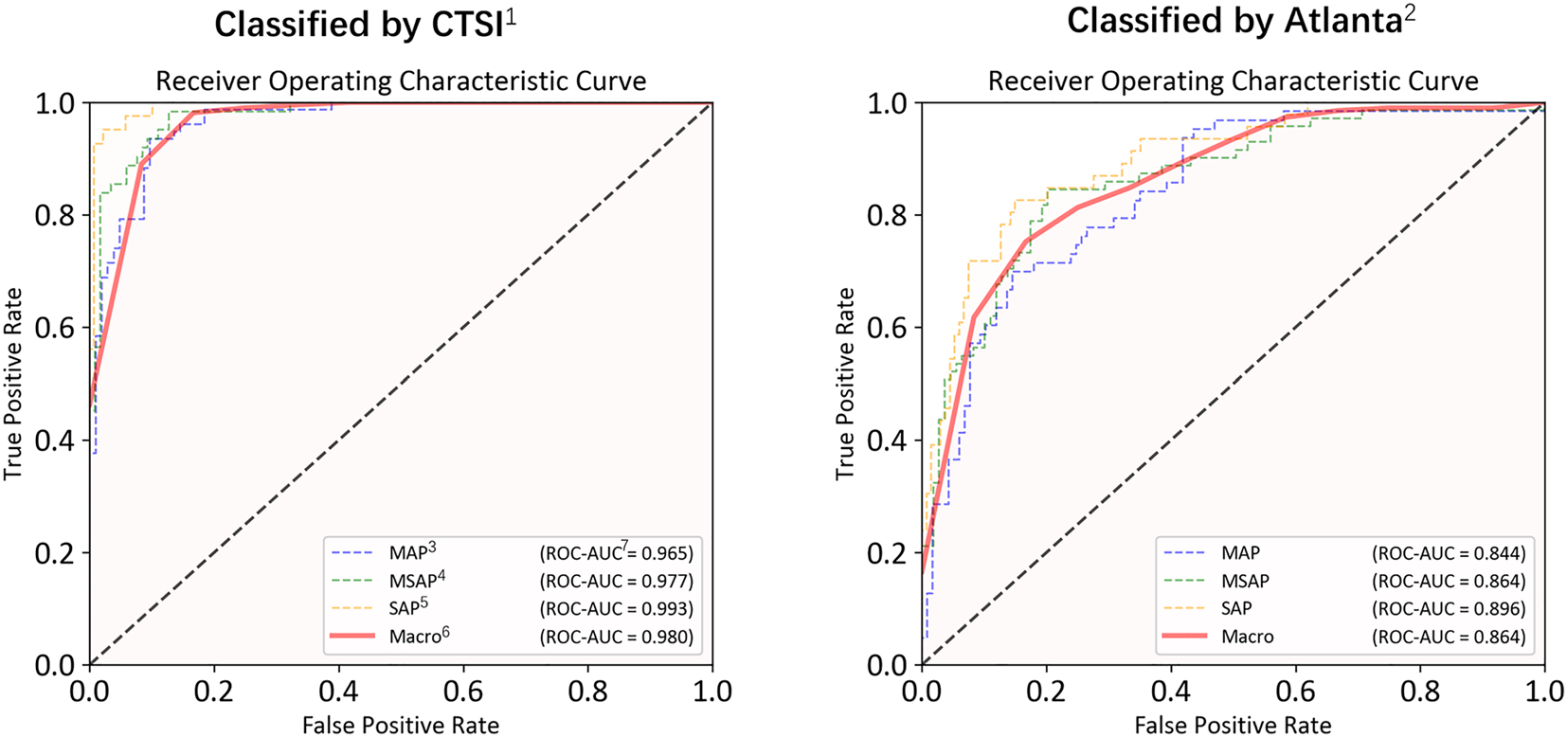
The receiver operating characteristic curves of the model prediction. Note: ^1^CTSI, computed tomography severity index; ^2^Atlanta, 2012 revised Atlanta classification of acute pancreatitis; ^3^MAP, severe acute pancreatitis; ^4^MSAP, moderately severe acute pancreatitis; ^5^SAP, severe acute pancreatitis; ^6^Macro, macro-average; and ^7^ROC-AUC, areas under the receiver operating characteristic curve.

The ROC curves of the DenseNet model predictions for the two different labeling methods are illustrated in Fig. 4. Both methods exhibited the best prediction performance for SAP, likely due to the more pronounced CT image changes observed in patients with SAP, making them more easily distinguishable by the model. Furthermore, the model demonstrated superior predictions with CTSI labeling compared to the Atlanta classification (macro-average AUC-ROC: 0.980 vs. 0.864, *P* < 0.05; macro-average F1 score: 0.835 vs. 0.670, *P* < 0.05).

**Figure 5:**
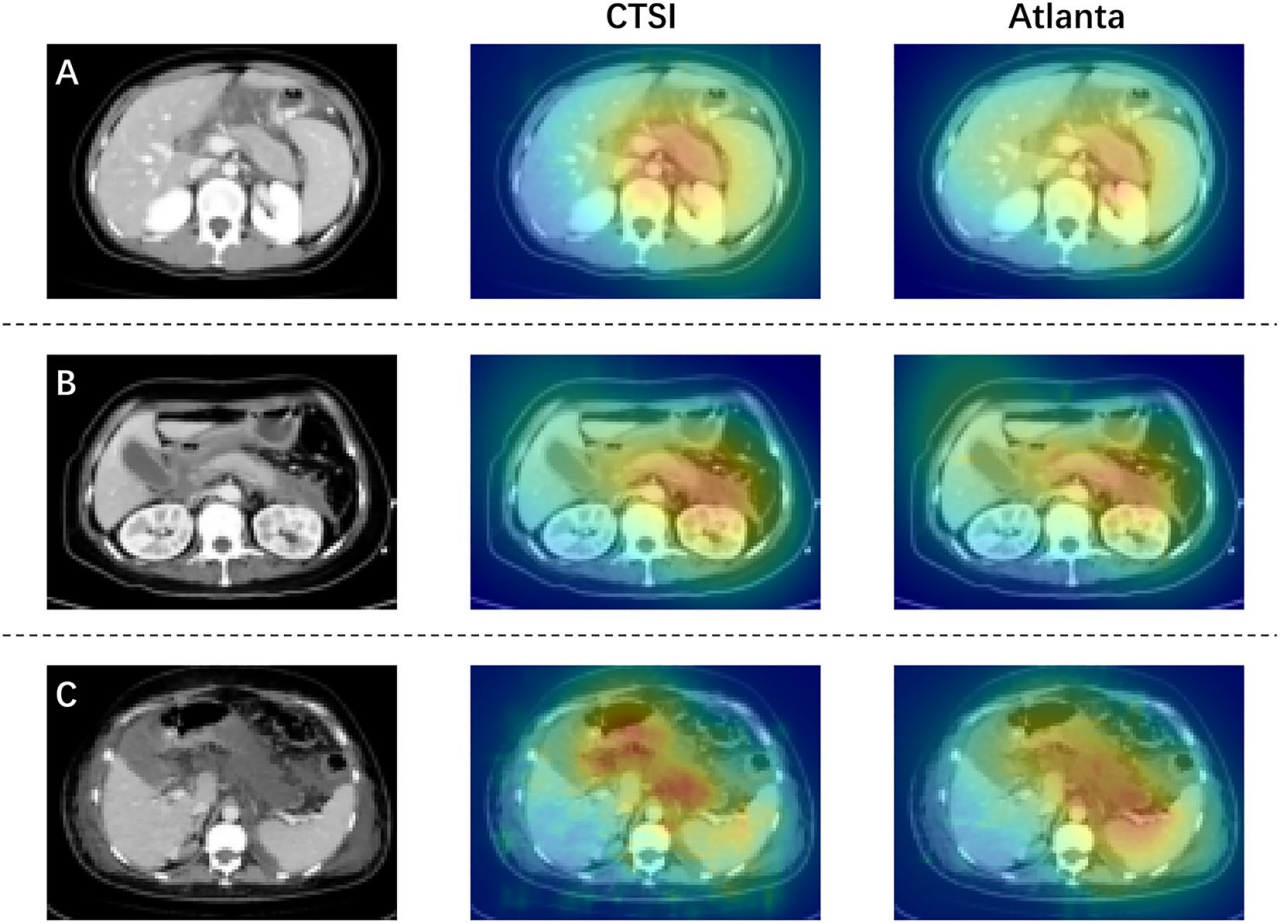
Visual explanations of model predictions using Grad-CAMs. The left column shows original CT scan images, the middle column displays visual explanations obtained from the model trained with CTSI-labeled CT scans, and the right column shows visual explanations from the model trained with Atlanta-labeled CT scans. (**A**) CT scan slice with mild pancreatic enlargement; (**B**) CT scan slice with peripancreatic accumulation; (**C**) CT scan slice with extensive pancreatic necrosis and peripancreatic accumulation.

The visualization results of our model can be seen in Fig. 5. We selected three representative CT scan slices of AP and employed Grad-CAMs to visualize the regions influencing the decision-making process in our trained DenseNet models. Our findings reveal that in the case of MAP with a pancreatic enlargement (Fig. 5A), both DenseNet models trained with the two annotation methods focused on the pancreas and the surrounding peripancreatic region. However, in instances with more noticeable pancreatic morphological changes (Fig. 5B and C), the DenseNet model trained using CTSI-labeled CT scans more effectively accentuated the areas corresponding to pancreatic necrosis and peripancreatic accumulation.

## Discussion

Our study confirms the feasibility of using the CNN models, grounded on 3D DenseNet, to predict the severity of AP using enhanced CT scans. Regardless of whether the models were trained on CT scans labeled CTSI or Atlanta classification, the trained model consistently yielded robust classification performance, with a macro-average AUC-ROC score surpassing 0.8.

In recent years, advancements in AI and deep learning have enabled the application of CNN models, such as ResNet, DenseNet, Inception, and VGG, in the automatic analysis of CT scans^30–33^. DenseNet has demonstrated favorable predictive performance in studies on the prediction of various conditions, including COVID-19^34^. Previous studies predominantly utilized 2D CT slices for training and testing CNN models^35^. However, since CT scans inherently provide 3D information, processing them using 2D models may lead to the loss of valuable information and compromise predictive efficacy. With the advancement of computing capabilities, the use of 3D models for direct processing of CT scans has become feasible. Studies involving the automated identification of COVID-19 patients have shown promising results using 3D CNN models^36^. Therefore, in this study, we employed a 3D DenseNet model based on these previous findings.

To the best of our knowledge, there is limited research on using deep learning models to predict the severity of AP from CT scans. In a recent study, Chen et al. employed an image-deep learning model based on MobileNetV2 and trained it on non-enhanced CT scans obtained within 72 h of onset^20^. In this study, we trained a CNN model based on 3D DenseNet using enhanced CT scans from patients with AP, typically taken around 5.4 days after symptom onset. The focus of the two studies varies, and the superior predictive performance of our models demonstrates the potential advantage of using enhanced CT scans for severity prediction of AP through CNN models, aligning with pancreatitis treatment guidelines and clinical experience. However, both studies preliminarily suggest the promising potential of CNN models in predicting the severity of AP using CT scans. However, there remains a considerable gap between the current capabilities of these models and their potential clinical applications, emphasizing the need for continued research.

The Atlanta classification is currently the most prevalent method for severity classification in AP. While there is a correlation between the CTSI and Atlanta classification, they are not identical^37, 38^. The primary rationale for employing the CTSI in our research stems from its exclusive reliance on CT imaging, which sets it apart from the Atlanta Classification which also factors in additional data beyond the scope of CT imaging. Given these distinctions, CT images may not carry sufficient information for a precise Atlanta classification, and the utilization of Atlanta classification labels in training the CNN model may compromise the model's generalization capability.

Indeed, in this research, we amassed as many enhanced CT scans of patients with AP as possible. Several patients underwent multiple CT scans, and if these scans met the study's inclusion and exclusion criteria, they were included. As pancreatic necrosis can develop during the early stages of AP, the CTSI classification for these patients may fluctuate over different time points. However, their classifications according to the Atlanta criteria remained consistent. Training the model on distinct CT images carrying identical labels may detrimentally impact the model's predictive capacity. Our results showed that the predictive performance of the model trained on CT scans labeled with CTSI outperformed the model trained on CT scans labeled with the Atlanta classification (macro-average AUC-ROC: 0.980 vs. 0.864). These findings indicated that training a CNN model using enhanced CT images based on the CTSI can achieve better predictive performance, and it demonstrates the ability of the CNN model to capture information about changes in the severity of AP from CT images.

In this study, we did not perform an a priori extraction of the region of interest (ROI) pertaining to the pancreas. Currently, there are no mature algorithms for accurately classifying pancreatic necrosis, peripancreatic necrotic accumulation, and normal pancreas^39^, and manual ROI labeling or training a separate model for automated segmentation would require substantial time and effort^40^.

One of the key advantages of deep learning models is their ability to automatically extract quantitative features from high-throughput images, analyze image data in-depth, and translate microscopic lesion changes into quantitative measures^41^. In this study, referred to some previous CNN model research^42–44^, we adopted an end-to-end approach that bypasses ROI extraction and directly employs the entire CT scan for model development. Fortunately, our results affirmed the feasibility and effectiveness of this direct approach in predicting the severity of pancreatitis. Regardless, employing an appropriate pancreatic segmentation algorithm or extracting ROI could potentially enhance the predictive performance of the model. This issue can be further explored in subsequent research.

To further evaluate whether our model effectively focuses on the pancreas and peripancreatic necrosis, we employed the Grad-CAMs visualization technique to highlight potentially decision-related areas in CT scan slices^27^. The Grad-CAM results confirmed that the model successfully attends to areas of both pancreatic and extrapancreatic necrosis, further supporting the model's applicability.

In the field of artificial intelligence for pancreatitis, one of the ultimate goals might be to dynamically predict the severity of a patient's condition and their clinical prognosis based on various collected data, thereby guiding clinical diagnosis and treatment. In future research, through model improvements, such as adopting attention-based transformer models and incorporating time-series based recurrent neural networks, we may not only further enhance the predictive performance of the model and reduce computational load, but also potentially achieve a dynamic evaluation of pancreatitis severity using heterogeneous data from different time points. This could enable us to rapidly and accurately determine the severity of AP using available data.

However, this study has certain limitations inherent to its design and objective conditions. Firstly, it was a single-center study, which limits its generalizability, although sample consistency was high. Conducting multicenter studies and external validation would further strengthen the predictive efficacy of the model. Secondly, as mentioned earlier, ROI extraction was not attempted in this study, and it remains unclear whether performing ROI extraction would improve the model's prediction performance. This aspect can be explored in future research. Thirdly, in this study, we focused on developing a 3D DenseNet CNN model. Future studies can investigate additional CNN models and cross-modal hybrid models that integrate both imaging information and clinical data to enhance the model's performance in predicting AP severity.

In summary, our findings demonstrate that the constructed 3D DenseNet CNN model exhibits reliable predictive capability in classifying AP severity after training with enhanced CT scans, highlighting the feasibility of using CNN models for automatic AP severity classification based on imaging data. Moreover, this study provides insights for the development of more comprehensive models that incorporate both imaging information and clinical data for predicting the severity of pancreatitis. Further advancements in this area can lead to improved clinical decision-making and better patient outcomes.

### Supplementary Information

Supplementary Information.

## Data Availability

The data generated and analyzed during the current study are not publicly available due to privacy laws and policies, but are available from the corresponding author on reasonable request.
