# Supplementary material for: Predicting acute pancreatitis severity with enhanced computed tomography scans using convolutional neural networks
Source: Sci Rep. 2023 Oct 16;13:17514. doi: 10.1038/s41598-023-44828-7 (PMC10579320; doi:10.1038/s41598-023-44828-7)
Supplement: Supplementary file 1 — Supplementary Information. [file 41598_2023_44828_MOESM1_ESM.docx]

We divided our sample based on the consistency of CT scan annotations into two groups: the consistent annotation group (n=1,625) and the inconsistent annotation group (n=173). We randomly selected 100 instances from each group to validate using our trained models. It should be noted that this validation is not entirely rigorous, as the training and validation datasets are not fully congruent. Therefore, we have included this portion of the analysis in the supplementary material.

The predictive performances of trained models using the sampling from these group are presented in the subsequent table. Regardless of whether sampling from the consistent annotation group or the inconsistent annotation group, the model trained using CTSI outperformed those trained with Atlanta classification. Notably, the disparity in prediction performance appears even more pronounced in the inconsistent annotation group sample. As discussed in other sections, the CTSI-based classification is determined by the current CT scan, while the Atlanta classification also considers the patient's clinical course and other factors. Employing a classification like CTSI, which more closely aligns with the current status of CT scans, might benefit the model in achieving more accurate predictions. This insight will guide our future research endeavors.

| Table. Predictive performances of trained models using the sampling from the consistent annotation group and the inconsistent annotation group | | | | | |
| --- | --- | --- | --- | --- | --- |
|  | Accuracy | Recall | Percision | F1 score | AUC-ROC |
| Model trained by CTSI^1^ |  |  |  |  |  |
| within consistent group | 0.897 | 0.811 | 0.904 | 0.841 | 0.972 |
| within inconsistent group | 0.844 | 0.798 | 0.879 | 0.840 | 0.962 |
| Model trained by Altanta^2^ |  |  |  |  |  |
| within consistent group | 0.811 | 0.586 | 0.792 | 0.667 | 0.841 |
| within inconsistent group | 0.751 | 0.516 | 0.778 | 0.610 | 0.784 |
| Note: All prediction parameters are macro-average metrics. ^1^CTSI, computed tomography severity index; ^2^Atlanta, 2012 revised Atlanta classification of acute pancreatitis. | | | | | |
